# Shared action spaces: a basis function framework for social re-calibration of sensorimotor representations supporting joint action

**DOI:** 10.3389/fnhum.2013.00800

**Published:** 2013-11-26

**Authors:** Giovanni Pezzulo, Pierpaolo Iodice, Stefano Ferraina, Klaus Kessler

**Affiliations:** ^1^Institute of Cognitive Sciences and Technologies, National Research CouncilRome, Italy; ^2^Department of Physiology and Pharmacology, Sapienza UniversityRome, Italy; ^3^School of Life and Health Sciences, Aston Brain Centre, Aston University, Aston TriangleBirmingham, UK

**Keywords:** joint action, perspective taking, basis function, sensorimotor transformation, spatial alignment, mental alignment, social interaction

## Abstract

The article explores the possibilities of formalizing and explaining the mechanisms that support spatial and social perspective alignment sustained over the duration of a social interaction. The basic proposed principle is that in social contexts the mechanisms for sensorimotor transformations and multisensory integration (learn to) incorporate information relative to the other actor(s), similar to the “re-calibration” of visual receptive fields in response to repeated tool use. This process aligns or merges the co-actors’ spatial representations and creates a “Shared Action Space” (SAS) supporting key computations of social interactions and joint actions; for example, the remapping between the coordinate systems and frames of reference of the co-actors, including perspective taking, the sensorimotor transformations required for lifting jointly an object, and the predictions of the sensory effects of such joint action. The social re-calibration is proposed to be based on common basis function maps (BFMs) and could constitute an optimal solution to sensorimotor transformation and multisensory integration in joint action or more in general social interaction contexts. However, certain situations such as discrepant postural and viewpoint alignment and associated differences in perspectives between the co-actors could constrain the process quite differently. We discuss how alignment is achieved in the first place, and how it is maintained over time, providing a taxonomy of various forms and mechanisms of space alignment and overlap based, for instance, on automaticity vs. control of the transformations between the two agents. Finally, we discuss the link between low-level mechanisms for the sharing of space and high-level mechanisms for the sharing of cognitive representations.

## Introduction

Goodale and Milner (1992) proposed a segregation into perception-for-identification (of objects) vs. perception-for-action and empirically corroborated this claim in later work relating the former to the ventral (occipito-temporal) and the latter to the dorsal (occipito-parietal) processing stream, respectively (Milner and Goodale, 2008). While the ventral stream seems to employ relative metrics based on an environment-/object-based frame of reference (FOR), the dorsal perception-for-action stream codes “real” distances within an egocentric FOR (Aglioti et al., 1995; Ganel et al., 2008). This distinction is crucial in the present context, where we will focus primarily on perception-for-action and the properties of the dorsal stream.

The way we organize and neurally represent the space around us in the dorsal stream is functional to action performance and not only to the description of where objects are (Goodale and Milner, 1992; Rizzolatti et al., 1997). During sensorimotor learning, the actions we perform shape our perceptual representations so that they support efficient sensorimotor transformations such as the calculation of the motor commands required to achieve a goal (e.g., reaching and grasping an object) and the prediction of the sensory consequences of actions (Wolpert et al., 1995; Pouget et al., 2002). These sensorimotor transformations are often (but not exclusively) linked to a brain network that includes the dorsal processing stream, i.e., the posterior parietal cortex (Colby and Goldberg, 1999; Ferraina et al., 2009a), the premotor cortex (Graziano et al., 1994; Rizzolatti and Luppino, 2001), yet, also the cerebellum (Wolpert et al., 1995; Kawato, 1999).

This tight relationship between visuo-spatial representations and actions implies that spatial locations must be encoded in relation to the instantaneous and multisensory internal representation of the agent’s body in order to account for the flexibility and precision of action execution, disregarding other aspects such as the particular body posture and limb locations in relation to the environment (Gross and Graziano, 1995).

This action-based view of visuo-spatial processing in the dorsal stream predicts that the neuronal mechanisms supporting spatial perception and multisensory integration should be dynamic. In this vein, Head and Holmes (1911) have proposed that the brain maintains and continuously updates a multimodal representation of the body: a *body schema*. During movement or learning of new motor skills, the body schema is updated to code where the body parts are located in space and what is their configuration. During development, the body schema is updated to code for new action possibilities due to growth or the acquisition of new motor skills. Furthermore, the body schema should incorporate action-relevant objects and thus be updated when using tools that (for example) extend the reach. For example, the visual response fields of bimodal neurons in monkey intraparietal area (modulated by both somatosensory and visual stimulation) expand as an effect of tool use to include the entire length of the tool (Maravita and Iriki, 2004). In other words, learning to use novel tools stretches the body schema or extends the internal representation of the actor’s hand (Arbib et al., 2009). Patients suffering of hemispatial neglect in their near space, as a consequence of parietal cortex lesions, display symptoms in the far space when using a tool to extend their action potentials (Berti and Frassinetti, 2000). Other studies showed that tool use also influences perceptual judgments; for instance, the egocentric distance to a target object is perceived smaller when holding a tool (Witt and Proffitt, 2008). These studies suggest that the dynamic aspects of multisensory receptive fields and perceptual representations depend on the execution of goal-directed actions, consistent with the idea of a common coding of perception and action in *ideomotor* theories (Prinz, 1997).

In this article we extend the principles of the action-based approach to the case of social interactions. We propose that co-actors engaged in social interactions and particuarly those having common goals (e.g., lifting together a table, playing beach volleyball as a team) are able to include other-agent’s operational spaces in their own space representation.

Numerous studies have shown that co-actors’ perception-action loops are not independent but can influence each other (Sebanz et al., 2006). This evidence can be interpreted using a non-representational framework that describes interacting agents as coupled dynamical systems (Kelso et al., 2013). Alternatively, it has been proposed that co-actors continuously use predictive mechanisms (e.g., forward models) to predict both one’s own and another’s actions, and successively integrate this information to form an action plan (Sebanz and Knoblich, 2009). The prediction of another’s action is often described in terms of an *action simulation* that reuses the same internal models as those implied in one’s own motor control (Blakemore and Decety, 2001; Wolpert et al., 2003; Jeannerod, 2006; Pezzulo et al., 2007, 2013; Dindo et al., 2011; Pezzulo, 2011a,b). This mechanism is plausibly a costly one, as it requires planning and controlling one’s own actions while at the same time simulating the co-actor’s (possibly using the same internal models for both control and simulation). Furthermore, simulating another’s actions requires an intermediate computational step (i.e., transformation) when the actors are not perfectly aligned in space: an egocentric “shift” from the observer’s to the observed FOR, which is often called *perceptual* (Johnson and Demiris, 2005) or *visuo-spatial perspective taking* (e.g., Zacks and Michelon, 2005).

While not denying the importance of the aforementioned mechanisms based on dynamic coupling and action simulation, we advance a theoretical proposal based on the idea that an agent performing a joint action could benefit from an additional mechanism, a neurally represented “Shared Action Space” (SAS), which directly incorporates information relative to the co-actor in one’s own mechanisms for space representation and sensorimotor transformation.

### Shared Action Spaces support joint actions

The basic proposed principle is that in social contexts the mechanisms for sensorimotor transformations and multisensory integration (learn to) incorporate information relative to the co-actor. As an effect, the mechanisms supporting spatial representations of both agents are re-calibrated, in analogy to the re-calibration of visual receptive fields due to tool use (Maravita and Iriki, 2004). Thus, the co-actors can perceive and act using a *SAS* (where the word “shared” is chosen in analogy with the idea of sharing cognitive representations during joint actions (Sebanz et al., 2006); see below for a relation between these phenomena).

The social re-calibration provides a useful ground for performing numerous computations required for joint actions; for example, remapping coordinate systems and FORs (e.g., from my-eye-centered FOR to a your-eye-centered FOR or even our-position-centered FOR), sensorimotor transformations (e.g., learning the movements and amount of force necessary to lift an object jointly with another agent), and motor-to-sensory transformations such as forward modeling (e.g., predicting the sensory consequences of a joint action). The social re-calibration might thus constitute an optimal solution to sensorimotor transformation and multisensory integration in joint action or more in general social interaction contexts.

A SAS is usually *extended* compared to the individual action spaces of the co-actors and includes subspaces where actors interact or use other motor potentials. The extension of the operational space supports joint actions requiring both *simultaneous* and *complementary actions*. Consider for example the case of two persons lifting a heavy object together and simultaneously. In this case, the SAS may include *social affordances* (e.g., lifting affordances) that are not available to any of the individuals, who would not be capable of lifting the object by themselves (see also Richardson et al., 2007).

**Figure 1:**
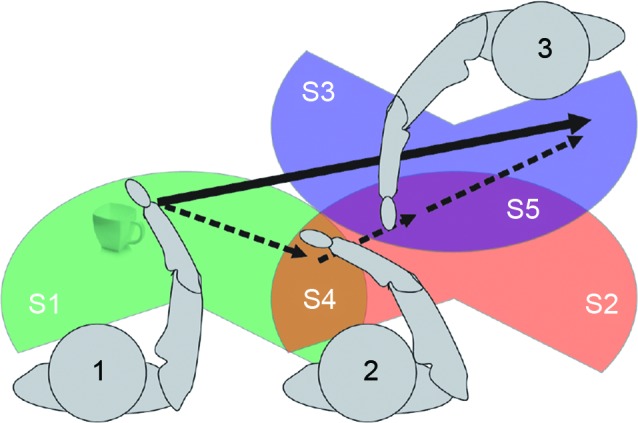
**A schematic illustration of SAS**.

As an example of complementary actions, consider a beach volleyball team of two players. The team can reach the ball everywhere within their half of the field even if each individual player can only reach a part of it; thus the group’s SAS is extended compared to the individuals’ operational space. Figure 1 provides a more detailed specification of the latter case. Three agents (1, 2, and 3) have their own operational space (S1, S2, S3, respectively) but also portions overlapping (S4 and S5) where agents could interact. The sum of S1, S2 and S3 represents the group’s SAS. Thanks to this space, it is possible for agent 1 to “move” the cup to the left side of agent 3 even if he cannot physically reach such location. To perform this action he has first to pass the cup to agent 2 (object in S4); subject 2 will then pass the object to agent 3 (object in S5) that, finally, will move the cup in the final position.

Operational spaces are of different significance. S4 and S5 represent “physically” SAS. In S4 both 1 and 2 could physically interact. The same is for S5 where the interaction is among 2 and 3. The extension and use of S4 and S5 depend on the inter-subject distance and relative orientation (both influenced by many factors; see below). However, for each of the subjects, the action space can be extended to the “virtually” SAS, even when direct interaction is not possible; for example, moving objects from S3 to S2 (or from S1 to S2) becomes an available option for all components of the group. If an agent (say, 1) neurally represents the virtually SAS, it can execute a single sensorimotor transformation to (plan to) move the cup from S1 to S3.

This example illustrates that groups such as those shown in Figure 1 have mixed ownership of space representations. Furthermore, the operational space of group members is extended. We propose that this phenomenon is produced by the neuronal mechanisms that support sensorimotor transformations, which are re-calibrated during social interactions. The re-calibration is similar to the extension of action possibilities due to tool use, except that the skills and action repertoires of the other group members are like “tools” that extend the individual action space into a SAS affording the achievement of individualistic and joint goals.

Note however that being physically close to other persons might not be sufficient to establish a SAS; it depends on the requirements of the situation as well as various social factors how (for example) S3 is merged into the shared space. If the action goal is to simply place the mug on the “far side” of S3 then the shared space would be a merged space as shown in Figure 1. If the goal is to place the mug on “left side” of S3 then at least agent 2 would need to represent S3’s left/right axis taking her orientation into account. Different situations might require other kinds of information such as the position, the line of sight, the goals or even the preferences and motor skills of the co-actor. Furthermore, since co-actors are not simple tools with only a passive role, social factors come into play such as the familiarity and trust of the co-actors in one another, as well the nature of the social interaction (say, cooperative vs. competitive) and the type of social context itself (e.g., informal vs. formal). Overall, then, various task and social requirements affect the way SAS are generated; see Section Prerequisites for Forming Shared Action Spaces and a Proposed Taxonomy.

The rest of the article is organized as follows. Section “Neuro-Computational Mechanisms Supporting Shared Action Spaces” describes the concept of SAS and proposes a neuro-computational mechanism for its implementation. Section “Prerequisites for Forming Shared Action Spaces and a Proposed Taxonomy” discusses the necessary preconditions for forming SAS and advances the idea that different mechanisms, based on automatic motor resonance or on deliberate embodied simulation, could be required depending on spatial relations and angular disparity alignment between the agents. Section “Socio-Cognitive Aspects of the Shared Action Space” discusses the relations between the idea of Shared Action Space and the sharing of cognitive representations and intentions.

## Neuro-computational mechanisms supporting Shared Action Spaces

The brain of living organisms receives information about the external world (e.g., the position of an object) from different sensory modalities (e.g., visual and auditory) and encodes them using different FORs, for example, eye-centered (i.e., distance between object and eye) for visual information and head-centered (i.e., distance between object and eye) for auditory information (Buneo and Andersen, 2006). Furthermore, information can be encoded in different coordinate systems; for example, the visual modality could encode the distance between object and eye in Cartesian (or polar) coordinates, centered at the eye or at other body’s parts (Lacquaniti et al., 1995). This multimodal information is spread in different brain areas; for example, it has been proposed that the parietal regions could use both eye-centered and hand-centered coordinates (Buneo et al., 2002; Ferraina et al., 2009b) and the premotor cortex could use body-centered representations (Caminiti et al., 1991; Graziano et al., 1994) or intermediate relative-position codes (Pesaran et al., 2006).

This information of the external world can be used to solve different problems in sensorimotor control. A first problem is *multisensory integration*, which consists in integrating information from different modalities to obtain a robust estimate of the position of the object, which in turn could require *coordinate transformation* and the remapping (or combination) of different coordinate frames. Still another problem is *sensorimotor transformation*, such as for example generating motor commands to reach and grasp the object (which in computational motor control is usually linked to *internal inverse models*). Solving this problem often requires coordinate transformations, too, such as when an eye-centered FOR used to visually locate the object has to be transformed in a body-centric or an object-centered FOR (representing the distance between the target object and the hand position and, finally, the effector shape) that could be more appropriate for reaching and grasping it (Jeannerod and Biguer, 1989). The opposite transformation (motor-to-sensory) is often required for the sensory prediction of action consequences, which in computational motor control is often linked to *internal forward models* (Wolpert et al., 1995).

**Figure 2:**
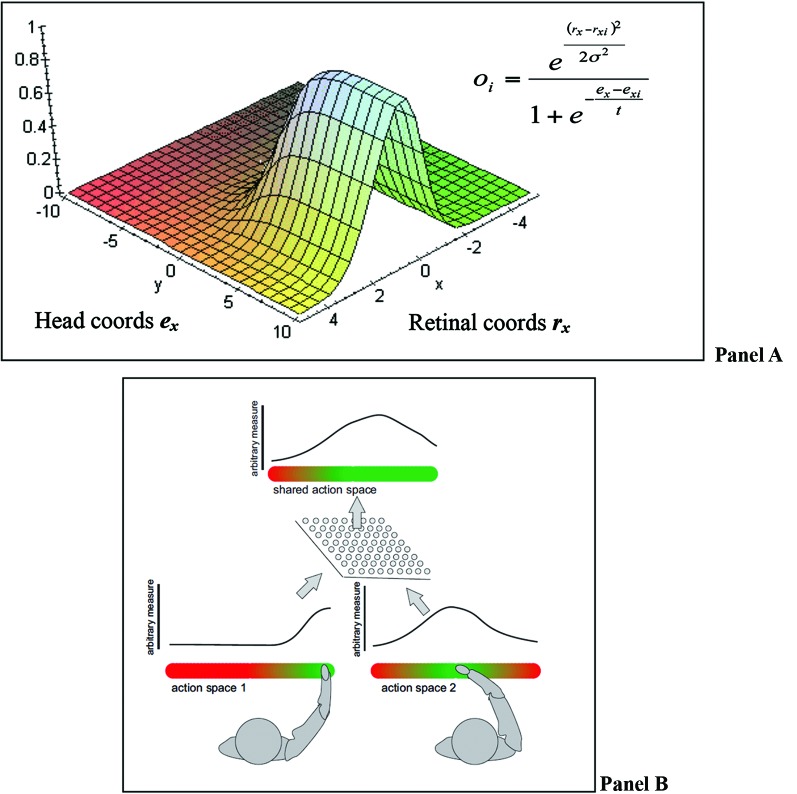
**(A)** BFMs permit combining different coordinate systems into a unique representation that encodes locations in multiple frames of reference; figure adapted from Kessler (2000). Panel **(B)** an equivalent BFM combining representations of different agents.

A recent computational theory of how the brain implements multisensory integration and sensorimotor transformations is the “basis functions” framework of Pouget and Snyder (2000) and Pouget et al. (2002). We adopt the “basis functions” framework to formulate our theory of SAS (but note that our theory can also be implemented differently and does not strictly depend on the basis function framework). In the basis function framework, all the streams of information are bi-directionally linked to a common basis function map (BFM; see Figure 2 Panel A).

The integration of signals at the level of the BFM (equivalent to an intermediate layer of a multi-layer network) permits solving sensorimotor problems using principles of statistical inference. It permits *coordinate transformation* because the BFM essentially encodes locations in multiple frames of reference simultaneously, creating a mixed FOR. It permits *multisensory integration* as multiple estimates (say of an object position) obtained by different sensory modalities (e.g., visual and auditory) can be combined in a mixed FOR and weighted by the relative reliability of the sensory modalities (e.g., visual information can be more reliable than auditory information).

There is indeed physiological evidence for such “combined representations” between inputs from different proprioceptive coordinate systems. Andersen and colleagues (reviewed in Andersen, 1994) reported neural populations in the macaque parietal cortex where the preference of specific neurons for a specific retinal location (i.e., the visual signal) was modulated by either head position lateral intraparietal (LIP) area or input form the labyrinth (area 7a). As a whole population such neurons have been proposed to encode combined maps as modeled by Pouget and colleagues (Pouget et al., 2002) as well as Andersen and colleagues (Andersen, 1994). What these results also suggest is that the egocentric perspective of an agent is the result of the non-linear combination of several proprioceptive FOR that encode locations simultaneously in eye-, head-, and body-related coordinates. For action-related coding limb-relative encoding of spatial locations could be particularly important and has indeed been reported in parietal area 7b of the macaque brain (Gross and Graziano, 1995).

Furthermore a basis function model proposed by Pouget and Sejnowski (1995) and Pouget and Sejnowski (1996) was able to explain a striking modulation of hemispatial neglect reported by Karnath et al. (1993). Karnath et al. showed that a stimulus in the affected hemifield could be perceived much more easily by neglect patients when they turned their body towards the stimulus. This revealed a direct modulation of eye-centred input by proprioceptive information about body posture in neglect, which was elegantly explained by Pouget and Sejnowski’s combined basis function model.

The basic architecture shown in Figure 2. Panel A also permits implementing efficient *sensorimotor transformations* (say reaching towards the object) not only because it supports the necessary coordinate transformations regardless of the sensory modality (e.g., from eye- or head-centric to body-centric FOR) but also because the BFM serves as an intermediate layer that permits approximating the *nonlinear* sensory-to-motor mapping as a combination of linear problems, see Pouget and Snyder (2000). As the information can flow in any direction (e.g., from sensory to motor but also from motor to sensory inputs), the same network permits also *forward modeling* and the prediction of the sensory consequences of actions.

**Figure 3:**
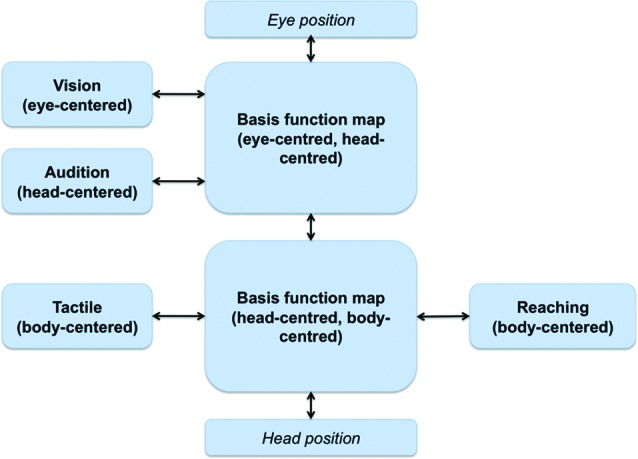
**Putative architecture supporting reaching actions; from Pouget et al. (2002).** Multiple stimuli dimensions, coded in different FORs, converge into BFMs that support sensorimotor transformations.

Figure 3 shows a BFN-based neural architecture supporting reaching actions that combines inputs from multiple (sensory and motor) modalities. Due to the bidirectional links, it supports transformations in all directions; for this reason, all the sources of information, either sensory or motor, can be considered both as inputs and outputs depending on the task at hand (e.g., a sensorimotor transformation from vision to action or a prediction from action to vision).

### From individualistic to interactive sensorimotor transformations

**Figure 4:**
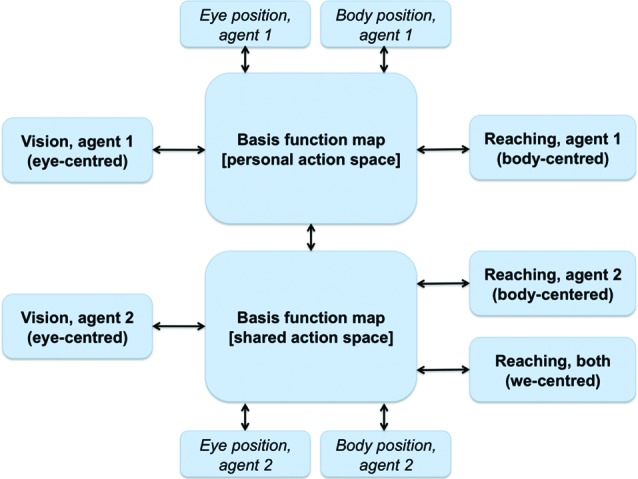
**A schematic representation of a BFM supporting perspective taking and joint actions.** See main text for explanation.

We argue that a similar architecture of combined basis functions can support joint action problems and the formation of a SAS between co-actors when information relative to the co-actor (e.g., its position, its actions) is linked to the BFMs. As shown in Figure 4, this can be achieved by extending the basis function idea of Figure 3. One possibility is that a single BFM can include sensory and motor modalities of oneself and another agent (e.g., one’s own and another’s eye, head and/or body positions). This map would support “individualistic” sensorimotor transformations (e.g., predict only the consequences of one’s own actions) when it only receives input relative to oneself. When it also receives inputs relative to another agent, the same network supports “social” sensorimotor transformations (e.g., predict the combined consequences of own and another’s actions). Another possibility, suggested in Figure 4, is that two separate BFMs code for individualistic and social sensorimotor transformations. In either way, the BFMs would come to encode a SAS in the sense that it simultaneously encodes the sensorimotor transformations of both agents, and beyond (e.g., actions that they can only do together such as lifting together a heavy object).

It is worth noting that sensorimotor transformations and remapping are predictive processes. For example, Duhamel et al. (1992) showed that receptive fields in LIP shift in the direction of saccades before the eyes have moved, and this mechanism maintains the visual scene stable. Similarly, sensorimotor transformations in the SAS are likely to be predictive processes about a co-actor’s future actions and how shared affordances may develop accordingly, which, in turn, is necessary for real-time coordination. In a similar vein, most theories of social interaction and joint action use the concepts of *action simulation* and *forward modeling* to emphasize that predictive processing is necessary for a correct unfolding of the interaction dynamics, see Pezzulo et al. (2013) for a review.

Note that all the associations shown in Figure 4 between the individual modalities and the BFMs) are bidirectional. This implies that not only the input modalities influence the BFM, but also vice versa, and so in principle an input can influence backward any other input. In “individualistic” sensorimotor transformations the bidirectionality creates subtle effects (some of which are empirically observed), including the fact that receptive fields linked to a given modality (say, auditory) can “shift” and the amplitude of their response changes when the inputs in any other modality change (e.g., when eyes are moved) (Pouget et al., 2002). This suggests the intriguing possibility that in the presence of SAS the coding of information relative to the others can influence one’s own multisensory coding. This possibility remains to be investigated in the future.

A potential problem with our proposal is that while one’s own body’s sensory and motor information is readily available through sensation and proprioception, the same is not true for information concerning a co-actor. However, several studies show that the boundaries of the body are not fixed and “bodily” representations can generalize and respond for example to the touch of a rubber hand (Botvinick and Cohen, 1998). Furthermore, there are various brain areas that encode “social” information, and which could give access to (at least a part of) a co-actor’s sensory, motor, and affective information, thus providing the kind of inputs required for our model. One possible source of information is the superior temporal sulcus (STS) that is implied in biological motion perception and could encode another’s visual and postural information (Saygin, 2007). Recently, Kessler and Miellet (2013) reported the so-called “embodied body-gestalt” effect (eBG), where the instantaneous posture of the observer directly impacts on how efficiently occluded bodies of other people are integrated into a body gestalt. This seems to suggest that proprioceptive information, i.e., the own body schema, directly impacts on the perception of another’s posture and actions, which could be mediated by combined representations in form of basis function networks. In extension of the eBG, physiological evidence exists for combined representations in the perception of space in relation to another’s body in form of visuo-tactile neurons that are sensitive to visual stimuli linked to another’s body (Ishida et al., 2010); see also Thomas et al. (2006).

Furthermore, mirror neurons could give access to information relative to another’s actions and their goals (Rizzolatti and Craighero, 2004). Mirror responses are sensitive to the operational space of perceived agents (Caggiano et al., 2009) and so could therefore signal the potentialities for interaction and the utility of integrating another’s actions into one’s own sensorimotor transformations (for example, for executing complementary actions). Mirror neurons are part of a wider “action observation network (AON)” that includes parietal, premotor, and occipitotemporal regions within the (human) brain and processes various kinds of information relative to other agents (Kessler et al., 2006; Biermann-Ruben et al., 2008; Grafton, 2009; Neal and Kilner, 2010). All this information is potentially relevant as an input dimension for forming the SAS (i.e., as one of the peripheral boxes of Figure 4). Furthermore, an intriguing possibility is that (portions of) the AON might constitute a proper part of the SAS itself rather than providing one of its inputs. If this is true, social resonance, mirror responses, and the body-gestalt effect could be reflections of the such combined representations (formalized here as BFMs and networks). Finally, resonance mechanisms (e.g., empathy for pain, Avenanti et al., 2005) could give access to another’s affective states that could be useful to modulate the sensorimotor interaction, see Section Problems and Open Issues of the Current Proposal.

It is worth noting that all the aforementioned processes act largely automatically. However, social cognition is supported by a range of deliberate mechanisms, too, which are often referred to as a “mentalizing” network (Frith and Frith, 2008). Although these mechanisms are typically associated with high-level information (e.g., inferring the beliefs of other agents) there are various demonstrations that they can influence social perception and ongoing action simulations, see Pezzulo et al. (2013) for a review. This suggests that an additional input can be provided by deliberate forms of perspective taking and embodied simulations that differ substantially from automatic effects. In Section Prerequisites for Forming Shared Action Spaces and a Proposed Taxonomy we elaborate on the idea that different kinds of spatial arrangements between the co-actors make some inputs, but not others, available, determining different characteristics of the SAS.

Overall, the mechanism shown in Figure 4 can integrate various aspects of the co-actor’s sensory, motor, and goal information (at least after proper training, see later). Although this information cannot be as reliable as one’s own proprioception, it could suffice to support efficient sensorimotor interactions and joint actions.

### How joint action problems are resolved within a shared action space

The SAS exemplified in Figure 4 provides a neuronal substrate permitting actors to co-represent the other agent(s) and to support joint actions (or more generally social interactions) efficiently. For example, it could permit perspective taking and the remapping of egocentric eye-centered coordinates between the co-actors (providing that an estimate of the co-actor’s position can be obtained). It could permit taking another’s movements into consideration when planning an action, which is useful for avoiding collisions but also for modulating one’s actions so that the combined effect with the co-actor’s actions is appropriate (say, when lifting a table together, the table remains stable and horizontal), or for calculating the combined operational space of the co-actors, as in the beach volley team example before. Below we discuss in detail how the SAS permits solving a few selected problems of joint actions and sensorimotor interactions.

#### Extending the operational space; multisensory aspects

As we have discussed before, experiments on tool use show that multisensory representations remap when new skills are acquired, suggesting that they code for an “operational space” that depends on action possibilities (e.g., how far I can reach) rather than absolute position of objects in space. The same multisensory remapping could occur as a consequence of the formation of a SAS, in which the action possibilities of co-actors (or more generally of agents engaged in social interactions) extend. For example, a somatosensory remapping could occur as a consequence of the extended operational space of a team of beach volleyball players; somatic and visual responses could be elicited that are linked to parts of the space that can be reached by any of the team. Figure 1 provides a schematic illustration of an extended operational space.

In analogy with the aforementioned evidence on tool use, it can be argued that every player sees the other players as “tools” that extend their bodies and action possibilities; for example, stretching the space that can be reached. A study conducted by Thomas et al. (2006) shows that sensory events can be elicited that are associated by the body of another person. The authors propose that such “interpersonal body representations” could be elicited automatically when seeing another person (thus, engaging in a joint action is not necessary).

The multisensory remapping could profoundly change the way we organize the space around us. A common distinction in spatial cognition is between *peripersonal* and *extrapersonal* space (Previc, 1998). Although different sub-divisions have been proposed, they are often described in terms of what actions they support (e.g., grasping space, ambient extrapersonal space as the space where visual inputs can be collected), that is in terms of operational space; see Rizzolatti and Luppino (2001). This conceptualization suggests the possibility that what is considered a peripersonal or an extrapersonal space changes as a function of social interactions; for example, the peripersonal space of a team of beach volleyball players could combine the individual peripersonal spaces with mixed ownership. In this case, the extended operational space consists of two peripersonal spaces with overlapping parts. Similarly, the extrapersonal space that normally is mapped by visual or acoustic modalities (but also olfactory; Koulakov and Rinberg, 2011) should be influenced by social interaction. A portion of the visual space hidden by an obstacle could be re-integrated in the internal representation of the extrapersonal space using information provided by co-actors.

#### Extending the operational space; motor aspects

Up to the moment we have discussed somatosensory remapping. However, extending the operational space also changes what affordances and action possibilities are available. Twenty years of research on mirror mechanisms have shown that monkeys and humans code for goal-directed actions performed by other agents in a flexible way (Rizzolatti and Craighero, 2004) and can consider several details including the operational space of the agents (Caggiano et al., 2009) and the possibility of complementary actions (Newman-Norlund et al., 2007; for review see Kessler and Garrod, 2013). Other studies suggest that humans can code for the action possibilities of other agents, too, and that objects can activate affordances both when they are in one’s own and another’s reaching space (Costantini et al., 2010, 2011a,b). This evidence can be linked to the idea of a SAS that is extended compared to the individual action space. The SAS sketched in Figure 4 is modulated by both one’s actions and another’s actions, and one’s affordances and another’s affordances.

This information, once coded in the SAS, can be used for performing joint actions. For example, a beach volley player can use the model shown in Figure 4 to predict whether or not a teammate will catch the ball and so prepare in advance a complementary action.

Note that in the beach volleyball example the operational space is the combination of the individual operational spaces. There are other cases in which the presence of two or more co-actors creates truly novel possibilities for action. Consider for example an agent facing the problem of producing the necessary actions (including body and arms posture, force, etc.) to lift a heavy object together with a co-actor. The object cannot be lifted by any of the agents, but can be lifted if both combine their efforts. A problem is how an individual agent can form a motor plan or predict the consequences of a joint action. If she can only use her internal models (e.g., forward models) without taking into consideration her co-actors actions, she cannot generate the sensory prediction that the heavy object will be lifted. However, if her sensorimotor transformations are based on a SAS, her/their forward model can consider the combined effects of her and the co-actor’s actions, and predict effects that cannot be produced by individual actions. In a similar way, a SAS could permit an agent to incorporate another’s motor acts (e.g., the force that she will apply) into his own plans and mesh them for more accurate control and prediction.

It is important to distinguish between action goals that are congruent between the agents (e.g., imitation of martial arts movements during practice), that are complementary between the agents (e.g., during standard dance), and that are competitive (e.g., during martial arts competition). For instance, these goals may directly influence how information about another’s action space is integrated into the egocentric basis-function map(s). That is, one could think of another modulation in form of a basis function (e.g., sigmoid as in Figure 2A) that would reflect space/action selection likelihood, thus, resulting in dynamically augmented vs. inhibited spaces and actions. These space/action landscapes could dramatically differ depending on goals that are congruent, complementary, or competitive. For example, when imitation of a movement is required the basis function would augment the same action as expected/observed in the other agent. For a complementary or a competitive joint action the identical action expected/observed in another agent would be suppressed while an appropriate complementary action (that could be defensive or aggressive in the competitive case) would be augmented. These examples illustrate that the functioning and even the coding of BFMs are highly task- and goal-dependent.

#### Multisensory integration

The mechanism shown in Figure 4 permits combining the action space of two (or more) individuals. In turn, this permits integrating perceptual and motor streams of two or more individuals, which might prove useful for example for state estimation. Consider the problem of estimating the position or trajectory of an object lying between two persons (say a ball in beach volleyball). An actor’s eye/head coordinates of the ball are mapped onto her body/hand coordinates for action. At the same time, these are combined with the action space of the other person forming a SAS. Within the shared space, sensory and motor information of the other person can be integrated as well that might help forming a more robust estimation of the ball trajectory or position. For example, an actor can use the co-actor’s movements (e.g., if she moves towards the ball or not) as an additional source of evidence for estimating the ball (actual and future) position.

#### Perceptual perspective taking and the remapping between frames of reference

As mentioned in the previous sections, the social context itself as input could have a direct modulatory effect on the combined representations in the basis function network(s) triggering a transition from an individualistic to a social or SAS. This may result in a combined operational space (a BFM of higher complexity, cf. Figure 4) or in a full switch to another action-guiding FOR; in other words *perspective taking*. In Section Prerequisites for Forming Shared Action Spaces and a Proposed Taxonomy we will describe in detail the spatial conditions under which perspective taking becomes necessary, while it is essential at this stage to point out the importance of the social context. In specific social situations, e.g., in a formal or hierarchical context such as a job interview, it is more likely that we adopt the other’s FOR (i.e., the interviewer’s perspective) than when chatting to a friend. Kessler (2000) proposed that such a direct influence of social context could also be represented as a combination of basis functions, where the likelihood of adopting the other’s FOR (or any other non-egocentric FOR) increases with the formality/hierarchy (see Tversky and Hard, 2009, for other dimensions) of the social context (cf. the eye/head model by Pouget and Sejnowski (1995, 1996) shown in Figure 2A, where “formality of social context” would be quantified on the y-axis and “FOR orientation” on the x-axis).

While social context could mediate the likelihood for adopting another’s FOR, the transformation process between the egocentric and the other’s FOR is a somewhat different matter. We propose that under specific circumstances, i.e., when people are spatially aligned the transformation between the egocentric FOR and the other’s FOR could be computationally equivalent to the usual re-mappings of coordinate frames (say from eye- to hand-centered) necessary for the individual to plan and control reaching and grasping actions (see next sections for details). Evidence indicates that such egocentric-to-egocentric remapping can give access to sources of evidence that are unavailable to any of the two original perspectives (Becchio et al., 2013).

In contrast to the case when people’s viewpoints are aligned, when their viewpoints are mis-aligned their operational spaces cannot be easily merged and an action-guiding FOR must be chosen or negotiated (see next sections for details). This could be the FOR of one of the agents but some joint actions could benefit from adopting a common allocentric (e.g., object-centered) FOR, where it could be easier to exert detailed control over the combined effects of actions (e.g., ensuring that a lifted table remains horizontal). Although it remains largely unknown what coordinate frames are used during joint action, evidence indicates that joint attention can change the FOR from an egocentric to an allocentric one (Bockler et al., 2011).

In either case the transformation of the egocentric into a mis-aligned target FOR (either the other person’s or an allocentic FOR) is not easily described by means of combined basis functions. However, recent evidence suggests that this transformation process could be a gradual transformation within the body schema map(s) of the perspective taker (Kessler and Rutherford, 2010; Kessler and Thomson, 2010; Kessler and Wang, 2012) that can be described as a shift within basis function networks. Kessler (2000) proposed a network model that used shifter circuits (Van Essen and Anderson, 1990) to shift the egocentric FOR orientation via intermediate orientations into the target orientation congruent to a simulated body rotation (Kessler and Thomson, 2010), which would be equivalent to the use of sensorimotor basis function networks in a “simulation mode”. That is, the anticipated sensorimotor and visuo-spatial outcomes are generated within the (individualistic) operational space by gradual orientation shifts without actually executing the usually associated movement. The result would be a spatially updated operational space with a simulated (egocentric) viewpoint as origin that would be spatially aligned with an allocentric or the other agent’s FOR.

### Problems and open issues of the current proposal

Despite its attractiveness, the basis function framework is computationally complex and prone to scalability problems; these problems could be magnified in social domains. Below we shortly discuss potential problems and open issues linked to our proposal.

An open issue is specifying how the computations linked to the SAS (e.g., the basis functions in the BFMs) are learned in the first place. In parietal cortex, mechanisms supporting sensorimotor transformations only arise after training and can be flexibly modified by new experience. In the same way, we propose that the SAS and in particular the basis functions required for the sensorimotor transformations are formed through learning. Humans and other social species often learn sensorimotor skills (e.g., lifting objects together with somebody other, playing volleyball) while engaged in social interactions and could acquire SAS as part of the sensorimotor learning. Of course the quality of the social skills and SAS depend also on the nature of the training; sensorimotor transformations can be more or less reliable when we play volleyball with our usual partners or when we interact with a stranger (the differences are also due to the success or failure of other mechanisms such as mindreading). Given that the computations of the basis function framework are hard even in individual domains, it is unclear if and how it can scale up to “social” sensorimotor skills. A scheme that is often used for scalability is making the architecture more modular. In this sense, it is possible to hypothesize that the formation of a SAS could require forming new BFMs specialized for social interactions rather than (or in addition to) reusing and extending existing ones. Testing these possibilities empirically is an interesting direction for future research.

Another open issue is what is the better FOR for performing joint actions such as lifting an object together or passing on an object. In some cases, a natural FOR can be the body position/orientation of one of the two actors (e.g., the actor who receives the object) (Tversky and Hard, 2009). This FOR permits controlling the action from the point of view of the receiving agent so that for example the “end-state comfort” (Rosenbaum et al., 2001) of the receiving agent can be optimized; as an example, the giver agent can pass an object to the receiver agent so that she grasps it comfortably (e.g., grasps a cup from the handle). In other cases, such as for example in symmetric joint actions (e.g., lifting an object together), an allocentric (object-centered) FOR can be used. Still another intriguing possibility is that joint actions benefit from creating novel “we-centered” frames of references, for instance a FOR that is centered between my body and your body, and novel metrics such as “relative to the distance between you and me” and “the sum of my force and your force”. The peculiarity of these metrics is that they are modified by the actions of both actors (e.g., the distance between you and me changes as an effect of my actions and your actions). They could be particularly efficacious for formulating some joint control problems, such as for example monitoring the distance between two volleyball players while performing a defence (Pezzulo, 2013). The fact that social groups (or teams) are hierarchically organized could further influence the form and extension of the SAS. A related problem is that it remains unclear so far, how different forms of spatial alignments and social requirements affect the selection or merging of individualistic FORs for establishing a common action space. This issue will be addressed in the next section where propose a taxonomy of SAS.

In the present model we are assuming that during social interactions agents perform with similar motivations. This is often untrue. One of the two volleyball players, in our example, could be more/less motivated during the match because of a larger/smaller expected personal reward. As a consequence, his influence on actions produced in the SAS will have more/less strength and the partner has-to/could adapt for optimal performance. Neural modulation for self and other reward outcome expectation/monitoring has been shown in different areas of the frontal lobe of primates (Chang et al., 2013) and the estimate of self/other motivational variables have been proposed to act as a gain modulation during common FOR generation (Chang, 2013). In this respect, a related issue to be considered is the level of each agent’s altruism, strongly influencing behavior, as revealed by all neurobiological studies exploiting game theory based approaches to decision making (Tankersley et al., 2007; Lee, 2008; Waytz et al., 2012). Because of these and other important factors influencing social interaction, the amount of shared space used by each individual and the number and contribution of actions to common goals are expected to be negotiable and more dynamic than what we are describing with our over simplification.

Finally, both the present model and most of the studies that explored action space of individuals and joint actions all dealt with agents unmoving. However, during a beach volley match every player changes his position continuously and so do the teammates. The same argument could be valid for describing synergic actions directed to objects that will change their position in space as a consequence of the cooperation. In all these cases, the SAS is dynamically updated in extension and boundaries in a non-easily predictable way. In this situation, a body-centered FOR of the action space could facilitate this continuous update of the representation of overlapped portions of the space more than an object-based or extra-personal FOR. Thus, our model is partial for describing all possible sources and forms of action space sharing and will require further aspects to be included in the future.

## Prerequisites for forming Shared Action Spaces and a proposed taxonomy

Up to now we discussed basic forms of integrating individualistic action spaces and hinted that different forms or mechanisms could be employed depending on social and spatial factors. One important distinction was made in relation to different action goals. We distinguished between action goals that are *congruent* between agents (e.g., imitation of martial arts movements during practice), that are *complementary* between agents (e.g., during standard dance), and that are *competitive* (e.g., during martial arts competition). These goals directly influence how information about another’s action space is integrated into basis-function maps, resulting in dynamically augmented vs. inhibited spaces and actions. While the goals differ, all these operations assume that the two action spaces can be directly merged into a shared space. However, direct merging might not always be appropriate and in the current section we will elaborate on the different *mechanisms* for combining spaces that define different types of SAS. Note however, that all shared spaces and combinatory mechanisms can be explained within the proposed basis function framework.

We propose a taxonomy that distinguishes between “*merged*” vs. “*aligned*” shared spaces, based on different social requirements and spatial characteristics of the interaction. This distinction is based on two main dimensions that characterize a joint action situation: (i) the social sophistication of the joint goal(s) and action requirements, in contrast to (ii) the spatial orientation/viewpoint difference between the two agents. The first dimension determines how much complexity and sophistication is required for one agent to represent the other’s experience of the world and their potential actions therein. The second dimension determines what mechanisms an agent can employ for mentally sharing an action space with the other (self-other mapping) depending primarily on the spatial layout between the two agents and their FORs (i.e., orientation difference) as well as other available FORs in the environment.

### Action requirements of a situation

It is important to distinguish between situations with low-level requirements for co-representation where individualistic action spaces can be combined via automatic resonance mechanisms (i.e., mirroring, e.g., Kessler and Garrod, 2013) or low level viewpoint matching, from situations with high-level requirements, where more explicit and controlled mental alignment is required (Kessler and Rutherford, 2010; Kessler and Miellet, 2013).

#### Low-level requirements (and Level-1 perspective taking)

As described in relation to Figure 1, the three agents might simply need to represent the overlap between their individualistic action spaces for placing the cup within “easy reach” of another agent. In general, situations like these would only require superimposing the egocentric and the other agents’ action spaces within a shared space, identifying areas of overlap. Another agent’s position, viewpoint or orientation in space matters only to the extent that it shapes their region of direct influence in relation to the egocentric space and those of any other agents. In these cases the individualistic action spaces can be directly merged according to the basis-function framework proposed above.

It is important to note that such low-level requirements and the associated merging of action spaces are also proposed to apply to the simplest form of perspective taking. Typically, perspective taking is regarded as a high-level, deliberate process of social cognition, yet, two different forms or levels of complexity have been identified (Flavell et al., 1981; Michelon and Zacks, 2006) and should be considered here. Level-1 perspective taking refers to understanding *what* another person perceives or not (e.g., what is visible to them or not), while Level-2 perspective taking refers to a deeper understanding of *how* another person experiences the world. The distinction is evidenced by different developmental onset ages (Level-1 ∼2 years; Level-2 ∼4–5 years) and cross-species differences, where certain forms of Level-1 perspective taking seem to be shared with other species, whereas Level-2 has so far been only conclusively identified in humans (Tomasello et al., 2005; Bräuer et al., 2007; Emery and Clayton, 2009).

This highlights the differences in complexity between the two levels, bolstering our argument that in situations where Level-1 perspective taking can resolve viewpoint/orientation differences, individualistic action spaces can be directly merged into a shared space. In the visual domain Level-1 perspective taking seems to be based on a mechanism that infers the line-of-sight of another agent based on their gaze information (Michelon and Zacks, 2006). In the present context and based on Pouget’s basis-function framework such a representation could be easily and directly transformed into body-related rather than head/eye-centred coordinates, allowing for judgments of “reachability” in addition to visibility. For instance, in a situation where it is only necessary to team off and grasp objects that are hidden from the other person’s view, then it is only important to represent the other’s line-of-sight to determine which actions will have to be performed by ourselves and which the other agent has available (Michelon and Zacks, 2006; Kessler and Rutherford, 2010). These two action spaces could be directly merged as no transformation is required beyond representing the others’ action space in relation to their body orientation and gaze direction; see Seyama and Nagayama (2005) for the integration between body orientation and gaze direction perceived in others. In general, coordinating actions that refer to very simple spatial relationships between agents and potential target objects will allow for direct merging of the agents’ action-spaces.

#### High-level requirements (and Level-2 perspective taking)

In contrast other social goals require more a sophisticated combination of action-spaces in form of alignment. This is the case for instance, when the spatial inter-relationship between agents and/or objects, such as “visibility”, are not enough but specific directional information (e.g., left vs. right) in relation to a particular origin or FOR is required. Specific mental transformations of the egocentric FOR of one agent into another are necessary in order to achieve such alignment (e.g., Kessler and Rutherford, 2010; Kessler and Thomson, 2010). The higher cognitive effort allows for more differentiated SAS where origin-specific directions can be distinguished and where the other’s body laterality is represented. For instance, one could directly determine if the other person uses their right or left hand/foot for an action. The default neurocomputational mechanism for the required transformation could be a simulated rotation of orientation in multiple basis-function maps, i.e., in multiple combined sensorimotor representations that constitute the internal body schema (e.g., Andersen, 1994; Pouget and Snyder, 2000).

**Figure 5:**
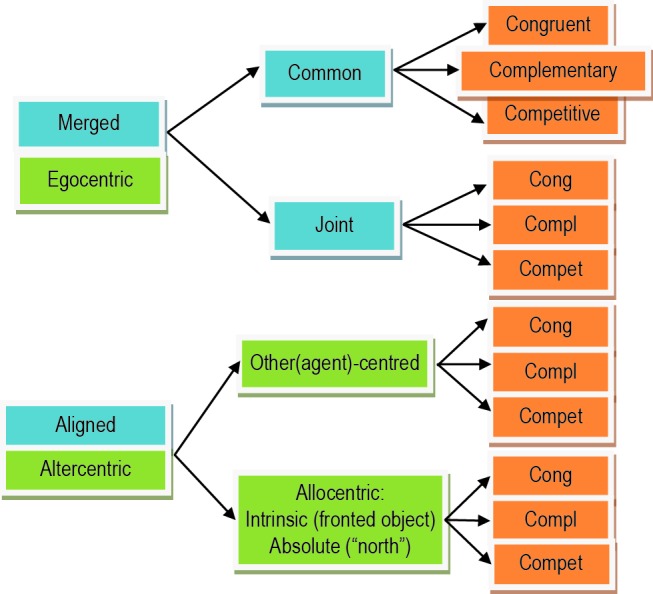
**Taxonomy for SAS divided into types (blue), origins (green), and action goals (orange).** SAS can be of the “aligned” or “merged” type, with the latter subdivided into “common” and “joint” subtypes. The origin of an SAS can remain within the egocentric FOR or can be transformed into another origin (“altercentric”) imposed by another agent (“other(agent)-centred”), an intrinsically fronted object (“intrinsic allocentric), or an absolute feature of the environment such as “north” (“absolute allocentric”). Goals can be “congruent”, “complementary”, or “competitive”. Further explanations are provided in the text.

Furthermore, if one agent mentally adopts another agent’s viewpoint for a more complex representational alignment, then this process can be congruent to Level-2 spatial perspective taking (Kessler and Rutherford, 2010). However, agents could also choose/negotiate to use neither of their FORs but a third, “allocentric” FOR instead and where both agents would have to accomplish a mental transformation into that FOR. Such a FOR could be in relation to a fronted object (e.g., the left or right side of a car), also called intrinsic allocentric or in relation to more absolute features of the environment (such as “north”), also called absolute allocentric (see Figure 5). For instance, volleyball players might not only represent a SAS relative to each other but in relation to the allocentric alignment of the playing field, thus, optimizing their SAS relative to the purpose of the game (i.e., they are typically facing the net and their adversaries). All these processes are usually strongly influenced by learning, after including in the own representation all potential sources of information useful for common goals. The transformation can be mechanistically congruent for alignment with another person or with an allocentric FOR and has been characterized as an embodied simulation of a body rotation. However, the social goals may substantially differ: alignment with an allocentric FOR would pursue the goal of imagining the self in that virtual perspective, in contrast to the goal of imagining another’s visuo-spatial experience in the case of alignment with another person’s FOR (see Figure 5).

Finally, disregarding which FOR is chosen in a given context, an embodied mental transformation into that FOR’s orientation only becomes necessary when the difference in orientation between the egocentric and the target FOR surpasses a certain angular disparity. This is where our second taxonomic dimension regarding spatial orientation differences ties in with our considerations so far.

### Spatial orientation/viewpoint differences between agents (and FORs)

The spatial/physical orientation difference between two agents can be crucial for how easy their action spaces can be merged. Merging refers to the direct integration of action spaces in the proposed basis function framework. If the two agents stand/sit next to each other, sharing a viewpoint, then their action-spaces can be easily merged disregarding the complexity of their joint goal—at all levels of complexity the mapping of their individualistic spaces into a shared space will be a direct merging operation. Nevertheless the complexity of the goal may determine what aspects of the action-space are represented at all (e.g., mere visibility vs. more sophisticated laterality). We propose to identify this case as the “*common*” shared space subtype of “merged” action spaces (see Figure 5).

If the angular disparity between the agents increases, then the effort of combining their action-spaces increases as well. Typically there is a discrete jump in cognitive effort (e.g., response times) at around 60–90° where the overlap between the two FORs diminishes (Kessler and Thomson, 2010; Janczyk, 2013). However, this increase in effort is *only* the case for joint action goals that require sophisticated spatial alignment (Kessler and Rutherford, 2010). In the case of simple goals, individualistic action spaces can still be directly merged, disregarding orientation differences, since actions are only constrained by origin-independent spatial relationships between agents and objects such as “visibility” and “reachability” (see Section Low-Level Requirements (and Level-1 Perspective Taking)). That is, action spaces can be merged directly even for agents being positioned face to face (=180° angular disparity). Figure 1 exemplifies this in form of S5 that defines the reachability overlap between Persons 2 and 3. As described earlier (see previous sections) merging operations are likely to rely on resonance mechanisms that automatically map the observer’s body repertoire (actions, postures) and instantaneous body schema onto an observed person (Kessler and Miellet, 2013). We propose to label this type of shared space as “*joint*” action space. The individualistic action spaces are merged, yet in contrast to a *common* action space, the agent’s viewpoints and orientations are not physically aligned.

In the case of complex goals, the two agents would have to settle on a particular FOR and mentally align their egocentric FOR with it to establish an “aligned” SAS. As proposed above, the default neurocomputational mechanism for the required transformation could be a simulated rotation of orientation in multiple basis-function maps, hence, the transformation can be resolved within the proposed framework. Once such a transformation into a common FOR has been accomplished there are at least two options for how this may affect the SAS. Note that we propose that a particular transformation indeed only needs to be conducted once for establishing the transition into the dominant FOR, but subsequently this FOR-dependent action-space will either replace the initially egocentric one or induce a specification of additional subspaces in a merged “*joint*” egocentric action space (e.g., my “left” is their “right” and vice versa) conform to the proposed basis function framework. Alternatively, however, several SAS might co-exist in concordance to the observation that several FORs can be simultaneously represented in typical (Furlanetto et al., 2013) as well as atypical neuro-cognitive processing (i.e., in heautoscopy, Brugger et al., 1997; Blanke and Mohr, 2005; Braithwaite et al., 2013). These are clearly hypothetical statements and further research is needed.

One exception to embodied transformation being the default mechanism at higher angular disparities (for complex requirements) may occur when the two agents are positioned face to face (=180° angular disparity). In this particular configuration agents may employ a different strategy by simply reversing their own egocentric space, for instance, “my left is your right” (Kessler and Wang, 2012). Again, this may feed into the specification of subspaces in a “*joint*” egocentric action space.

In summary we propose that socially shared space is not unitary and the following main features of the social and spatial configuration have to be taken into consideration for the way individualistic action spaces are combined into a shared space: (1) Below 60–90° of angular disparity between agents, merging into a SAS with a common egocentric FOR could occur directly, disregarding complexity of social requirements; (2) Angular disparities above 60–90° together with low-level requirements (e.g., “reachability”) may still be based on direct merging into a joint egocentric action space; yet, this egocentric FOR is not in common with the other agent; (3) Angular disparities above 60–90° together with high-level requirements (e.g., precise left/right distinctions) necessitate a transformation of the egocentric body schema into the orientation of another agent or into a common allocentric FOR in order to achieve an aligned action space with a common FOR. Strategies other than embodied transformation are possible, e.g., mental calculation (“my left is your right”) at 180°.

### Finalizing and exemplifying the taxonomy

We initially distinguished between action goals that are congruent between agents (e.g., imitation of martial arts movements during practice), that are complementary between agents (e.g., during standard dance), and that are competitive (e.g., during martial arts competition). These goals directly influence how information about another’s action space is integrated into basis-function maps, resulting in dynamically augmented vs. inhibited spaces and actions. Based on the above considerations we propose the following taxonomy of SAS. Primarily, we suggest distinguishing between merged vs. aligned shared spaces. While merged action spaces remain basically egocentric but are extended to incorporate the other’s action space, aligned spaces require a mental transformation into another FOR. In addition, for merged spaces we propose two further sub-types, resulting in three types overall (see Figure 5).

Firstly, a merging process may result in a common action space, which is the likely outcome when the agents are spatially/physically aligned (i.e., identical viewpoints). The resulting SAS can be directly described within the proposed basis-function framework. Common action-spaces could easily represent simple as well as sophisticated action requirements (e.g., place the cup into another’s “visible” vs. “right” space) since there is little or no discrepancy between individualistic FORs.

Secondly, joint action-spaces could be classified as spaces that have been directly merged despite strong orientation/viewpoint differences between the agents and their FORs. This is only possible with rather simplistic joint goals that only require determining “reachability”, “visibility” or other simple agent-to-object and agent-to-agent relationships (e.g., Level-1 perspective taking). Joint spaces can be directly represented within the proposed basis-function framework (cf. previous sections).

Thirdly, we propose that aligned action-spaces should denote combined spaces that have not been merged in a strict sense, but where, for instance, a dominant target FOR has been negotiated, which is shared between the agents (either one of the agents’ FORs or another intrinsic- or absolute-allocentric FOR). These action-spaces are likely to emerge in relation to sophisticated goals and interactions, requiring complex co-representation of another agent’s experience of the world and their potential actions therein (i.e., Level-2 perspective taking). The transformation into alignment is effortful and has been characterized (Kessler, 2000) as a simulated change of orientation within multiple combined sensorimotor representations (i.e., networks of basis-function maps) identified as the body schema that constitutes the egocentric FOR (Andersen, 1994; Pouget and Sejnowski, 1995, 1996). Thus, aligned action spaces can also be described within the basis-function framework; albeit, as a transformation- rather than a merging operation. Also note that after establishing FOR alignment, the resulting sub-space characterization could be used as input to specify a joint action-space within the basis-function framework, thus, not requiring further effortful transformation. Hence, it may well be that aspects of all three types of shared spaces could dynamically contribute to a single interaction, especially if more than two agents are involved (cf. Figure 1). To re-iterate, there is also the very interesting possibility that several shared space representations may co-exist simultaneously (e.g., joint and aligned) according to the observation, for instance in heautoscopy (for reviews see Blanke and Mohr, 2005; Furlanetto et al., 2013), that several perspectives or FORs may be represented in parallel.

Accordingly, the agents’ configuration in Figure 1 can be interpreted in different ways. Firstly, if the joint goal is to simply transfer the cup to the far side of Agent 3, then all three action spaces S1-S3 could be directly merged into a SAS. Note, however that each person would represent the other two in different ways, thus their shared space representations will differ, yet, for successful completion a few important aspects would be “meta-shared” (meaning that two or more agents have congruent representations in this respect), such as the overlapping action spaces (S4, S5). In this particular example Agent 1 would only need to (represent and) place the cup into S4, then Agent 2 would need to (represent and) take the cup from S4 and (represent and) pass it into S5, where Agent 3 (represents and) takes the cup, finally placing it into her egocentric left subspace of S3. Note however, that Agents 1 and 2 share their orientation, so their merged action space (including the overlapping subspace S4) is a *common* space, while Agents 2 and 3 merge their action spaces into a *joint* space as they are oriented face to face. Thus, their individual representation of the joint action space will have different origins, based within each agent’s egocentric orientation, however, this would not affect actions in relation to the overlapping space S5 as long as the joint action requirement remains simple (e.g., “placing the cup within reach”).

Secondly, for more sophisticated inter- and joint-actions the individualistic action spaces would have to be merged or combined in more sophisticated ways that specify more detail about subspaces. Agents 1 and 2 are physically aligned in space and would therefore generate a common shared space for substantial parts of the space surrounding them: The left side of S1 is to the left of both agents while the right side of S2 is also to the right of both agents. However, the quite crucial space in-between the two agents, S4, is ambiguous with respect to left/right labeling. The agents would have to determine that this subspace has opposite labels for each agent (i.e., “right” for S4 vs. S1, but “left” for S4 vs. S2) and include these into the shared space. According to our taxonomy the resulting shared space would then be a mix between a common and a joint space.

Similar considerations apply to Agents 2 and 3. Here the orientations differ dramatically (180°), so their entire action spaces (S2 vs. S3) will have opposite left/right labels. Again a mental calculation could quickly determine this opposite labeling and include these as subspace specifications within a joint action space (“left” within S2 is “right” within S3 and vice versa). Alternatively, at greater expense, one of the agents (e.g., Agent 2) could adopt the other’s perspective (Agent 3) and mentally align her action space with the other’s egocentric FOR. This would result in an aligned action space with the same origin for both agents (centred on Agent 3) and with identical left/right labels for both individualistic action spaces (S2 and S3). Such abstract considerations become highly relevant in particular social contexts. For instance, if Agent 3 is a child who is not yet very skilled in grasping a cup and/or the content is hot, then Agent 2 (e.g., the mother) might anticipate more precisely where and how to place the cup within S5: Placing the cup in the child’s “right” space with the handle turned towards the child’s right hand, would significantly facilitate the child’s task, yet, make it considerably harder for the mother in terms of specifying the child’s “right” subspace (which is actually the mother’s egocentric “left”) within a joint or an aligned SAS.

## Socio-cognitive aspects of the Shared Action Space

It may require extensive practice to generate SAS that lead to successful execution of joint actions. The mother and child example may only require the mother’s ability to conduct perspective taking and the child’s ability to grasp a handle for maximising the chances of success. Other joint actions require shared action plans that have to be extensively practiced alongside individual skills in order to maximise success. This would be the case for a beach volleyball team where the two players would learn to represent the other’s action space in relation to their own and to the playing field. Furthermore the two SAS representations that each player generates would need to have substantial features in common for avoiding misunderstandings, collisions, etc. Hence, practice will have to improve their individual playing skills, their representation of the other’s actions in a SAS, as well as the compatibility of their SAS at meta-level.

Up to now we have primarily considered perceptual, spatial and action-related determinants of SAS, such as the relative position of the two actors. Additional aspects such as the exact goals, action requirements and the social context play a crucial role but have only been assumed so far. However, it is likely that the formation and use of the SAS depend on socio-cognitive determinants such as the level of trust between the agents, group membership (in-group vs. out-group), etc. For example, recent evidence indicates that social exclusion is a determinant of action co-representation (Ambrosini et al., 2013; Costantini and Ferri, 2013).

Which FOR is chosen as the common, action-guiding FOR of a SAS can therefore depend on a variety of context factors such as the social relationship between the agents (e.g., hierarchy), the bodily ability for action (e.g., skill level, injury), or general characteristics of the social situation (e.g., “formality” of the situation as described in previous sections). Resuming our beach volleyball example, the SAS would differ if both players were equally good compared to when one player would clearly be the lead player, or if one player was a child or a learner, or if one player had suffered injury, e.g., was playing with an incapacitated arm affecting their action space on one particular side. It is also quite easy to imagine that SAS in this example would be quite different if it was a competitive game compared to more leisurely play.

In social structures with strong hierarchy, subjects tend to asymmetrically use their peripersonal/personal space. In military interactions, high-ranking agents use (move in) relatively more space than low-ranking agents (Dean et al., 1975). Signal integration is also influenced by social interaction. Heed et al. (2010) showed that the level of multisensory integration in peripersonal space is influenced by others actions in the same space and use of sensory signals.

We have used the example of tool-use to introduce the augmented space representation that usually follows agent-to-agent interaction. However, we are aware that a tool could only assume a passive role; there is no level of cooperation or interaction that could be described in tool-use. Thus, an important difference to tool-based extensions of an action space is that in agent-to-agent interaction spaces continuity of “force transmission” is not always important. In other words, tools but not other agents need to be physically manipulated. In the example of Figure 1, Agent 1 and Agent 3 have a common goal and successfully collaborate, both accessing the motor repertoire of Agent 2, without physically sharing parts of their peripersonal space. Recent research suggest that action co-representation between agents (Sebanz et al., 2003) also emerges when actors are positioned in different rooms but believe they are collaborating (Tsai et al., 2006). Thus, physical interaction may not be a necessary condition for social collaboration and SAS but it seems to facilitate sharing in specific experimental scenarios, see e.g., Guagnano et al. (2010). Finally, it should be noted that social information has various levels of complexity and some subjects could only be able to share some of it. Autistic subjects have difficulties with sharing high-level social information; in particular with all functions included in so-called “theory of mind” (Baron-Cohen, 2000), however they display normal access to low-level social information (Sebanz et al., 2005).

## Conclusions

Although it is well known that agents can have their abilities augmented by acting together with others, it in unclear how the brain mechanistically implements this process. Several mechanisms have been proposed that include entrainment, mutual prediction (Wilson and Knoblich, 2005), the sharing of representations (Sebanz et al., 2006), and a collective, we-mode of representation (Gallotti and Frith, 2013). In this article we argue that (at least some forms of) social interaction and social cognition (including cooperative and competitive ones) might be supported by the “social” re-use and re-calibration of the neuronal mechanisms for sensorimotor transformations and multisensory integration (Pouget et al., 2002).

We propose a *basis function* framework for social recalibration of sensorimotor representations; the resulting SAS are an embodied basis for joint action and sustained spatial and social perspective alignment. Coding the extended operational space and the social affordances created by the presence of co-actors in terms of *basis functions* for one’s own, another’s and joint actions could constitute a parsimonious solution to most interaction problems. This is especially evident if one assumes an ideomotor theory in which actions are coded in terms of their distal effects (Hommel et al., 2001). Co-actors sharing or merging their operational spaces can plausibly better plan, achieve, and monitor their joint goals. Future research would have to empirically assess our claims and in particular the proposed neuronal coding supporting “social” sensorimotor transformations that we have putatively identified as BFMs.

A second important direction for future research is understanding if and how the mechanisms that we described for *spatial* perspective taking can be considered as an example from which we can extrapolate to other, more complex forms of perspective taking and social cognition. Indeed, there are various demonstrations that during social interactions and in particular joint actions co-actors share representations and “align” at multiple levels, besides purely spatial alignments; some examples are mimicry of behavior, sharing of cognitive representations and formation of a linguistic common ground (e.g., during linguistic exchanges) (Clark, 1996; Bargh and Chartrand, 1999; Sebanz et al., 2006; Garrod and Pickering, 2009).

Spatial and cognitive forms of alignment have several similarities and could use similar computational principles (although not necessarily the same neuronal mechanisms). For example, a key aspect of the SAS is that it can be used for both planning one’s own actions and predicting another’s actions. The same feature is usually attributed to the common ground that is established during linguistic conversations (Clark, 1996) and to shared representations during joint actions (Sebanz et al., 2006).

Furthermore, we have emphasized that the SAS supports the alignment of individual FORs; one example would be the selection of a FOR centred on the body of the receiving person during a handout action. In a similar but more simplistic way, automatic mechanisms of resonance and mutual emulation are often advocated for the alignment of behavior (Bargh and Chartrand, 1999; Kessler and Miellet, 2013) and other forms of sharing and alignment (Garrod and Pickering, 2009), which in turn facilitate coordination.

In addition to automatic mechanisms co-actors can also adopt intentional strategies to form or calibrate a SAS. For example, co-actors (or a teacher and a student) can align spatially so that their operational spaces optimally overlap and the sensorimotor transformation does not require a complex rotation. In a similar way, intentional strategies of *signaling* help aligning the individualistic representations-for-action and “negotiating” a common plan for action (Pezzulo and Dindo, 2011); for example, a volleyball player can exaggerate her movements to signal a teammate that she is doing a left pass. Common ground formation during linguistic exchanges can help aligning the interlocutors’ situation models, which in turn facilitate the interaction. Future studies would be needed to assess if all these processes link to our proposal of SAS.

This discussion suggests that spatial and cognitive forms of perspective taking are not disconnected but rather have bidirectional influences. In this vein, Spivey (2012) has argued that the spatial intersection of individuals is always also an intersection of minds, because portions of shared space are occupied by another cognitive agent whose cognitive states can intersect with one’s own.

However, it is still unclear what are the mechanisms regulating the interactions between sharing action space and sharing cognitive representations. One simple explanation is that the mechanisms regulating spatial alignment and those regulating cognitive and affective evaluation (e.g., beliefs, liking, trust) of other persons are both regulated along the similar “positive—negative” dimension, and this can create positive feedback loops. For example, sharing a spatial operational space (or performing a joint action) can improve the positive beliefs (or affective reactions) and increase the trust in another person. In turn, because the persons now trust more one another, they come closer to one another and this in turn facilitates the sharing of their action space. The same mechanism can produce distrust and prevent the sharing of action spaces in other (e.g., competitive) situations. The plausi­bi­lity of this hypothesis remains to be assessed empirically.

## Conflict of interest statement

The authors declare that the research was conducted in the absence of any commercial or financial relationships that could be construed as a potential conflict of interest.
